# Recent advances in the roles of exosomal microRNAs (exomiRs) in hematologic neoplasms: pathogenesis, diagnosis, and treatment

**DOI:** 10.1186/s12964-023-01102-7

**Published:** 2023-05-01

**Authors:** Faride Nam Avar Jahromi, Razieh Dowran, Reza Jafari

**Affiliations:** 1 Department of Hematology, School of Paramedical, Shiraz University of Medical Sciences, Shiraz, Iran; 2 Department of Virology, School of Public Health, Tehran University of Medical Sciences, Tehran, Iran; 3 Research Center for Clinical Virology, Tehran University of Medical Sciences, Tehran, Iran; 4 Cellular and Molecular Research Center, Cellular and Molecular Medicine Institute, Urmia University of Medical Sciences, P.O. BoX: 1138, Shafa St., Ershad Blvd., 57147, Urmia, Iran

**Keywords:** Biomarker, Drug delivery, Hematologic neoplasms, Exosomal miRNA, Immunotherapy

## Abstract

In clinical diagnosis, the capability of exosomes to serve as biomarkers is one of the most important biological functions of exosomes. The superior stability of exosome biomarkers makes them superior to those isolated from traditional samples such as serum and urine. Almost all body fluids contain exosomes, which contain proteins, nucleic acids, and lipids. Several molecular components of exosomes, including exosome proteins and microRNAs (miRNAs), are promising diagnostic biomarkers. These exosomes may carry genetic information by containing messenger RNA (mRNA) and miRNA. The miRNAs are small noncoding RNAs that regulate protein-coding genes by acting as translational repressors. It has been shown that miRNAs are mis-expressed in a range of conditions, including hematologic neoplasms. Additionally, miRNAs found within exosomes have been linked with specific diseases, including hematologic neoplasms. Numerous studies suggest that circulating exosomes contain miRNAs similar to those found in parental cancer cells. Exosomes contain miRNAs that are released by almost all kinds of cells. MiRNAs are packaged into exosomes and delivered to recipient cells, and manipulate its function. It has been recognized that exosomes are new therapeutic targets for immunotherapy and biomedicine of cancers. The current review discusses the current evidence around exosomal miRNAs involved in the pathogenesis, diagnosis, and treatment of hematologic neoplasms.

Video Abstract

**Supplementary Information:**

The online version contains supplementary material available at 10.1186/s12964-023-01102-7.

## Background

Hematologic neoplasms are a wide range of diseases that any age group can be affected by this disease that originates from the bone marrow and lymph nodes affecting individuals of any age. Globally, lymphomas, acute and chronic leukemia, and multiple myeloma (MM) are the deadliest cancers. According to estimations, 176,200 people were exposed to various types of leukemia, lymphomas, or myeloma in 2019, accounting for 10% of all new cancer cases reported in the United States in 2019. They also accounted for 9.4% of 606,880 cancer deaths in 2019 [1].

Exosomes are a group of small membrane vesicles that are released by tumor or non-tumor cells into body fluids or the extracellular environment. These vesicles play a central role in cell communication through transfer between donor and recipient cells [2]. The lumen of exosomes contains various components such as DNA, RNA, lipids and proteins, which represent bioactive molecules in donor cells. MiRNAs are one of the exosomes cargoes that play a role in various cancer processes such as angiogenesis and metastasis [3].

The first evidence of the presence of miRNAs in exosomes was described by Valadi et al., in 2007 [4], reporting that exosomes contain miRNAs that can be transferred into acceptor cells and play relevant functional roles [4].The term microRNAs refers to a group of non-coding RNAs ranging from 19 to 25 nucleotides with prominent biological features [5–8]. The first miRNA was identified from a nematode *Caenorhabditis elegans (C. elegans)* in 1993 and linked to the organism's growth and development [6]. Seven years later, another miRNA, Lin-4, was discovered in *C. elegans*. That year (2000), humans' homologous let-7 was discovered, and more miRNAs were recognized soon after.The detecting procedure is still in progress [9]. According to recent theories, miRNAs affect the expression of one-third of human genes. Its ability to regulate numerous RNAs (mRNA) results from the fact that miRNA does not require a complete match to the 3' untranslated regions (3'UTRs) of messenger RNA. A recent study suggests that miRNAs select target mRNAs by interacting with the 3'UTR [10].

In recent years, a great deal of attention has been given to miRNAs in molecular pathway verification. As a result, some recent attempts have been made to use miRNAs as diagnostic biomarkers found in blood or plasma. Because exosomes possess unique characteristics, such as encapsulating miRNAs, being stable in circulation, reproducible, and reflecting cancer cells' characteristics. Highly selective diagnostic strategies may be developed to monitor cancer patients' condition rapidly and noninvasively through exosomes. However, no consensus has been reached yet on which miRNAs should be used at this time [11]. Exosomes are initially thought to serve as cell waste disposal containers, but exosomal miRNA extracts are currently preferred as diagnostic biomarkers due to their stability in serum/plasma. MiRNAs are also indicative of malignancies and pathological alterations in individuals. The exosome is a "bioactive vesicle" that mediate communication between cells and immune-regulation which shuttles molecules between cells [12, 13].

Exosomes, however, are believed to play an important role in physiological and pathological processes and contribute to cell-to-cell communication. Exosome compositions have also been linked to specific diseases and therapeutic effects. Thus, exosomes can be used to diagnose diseases. The serum or plasma levels of miRNAs are not correlated with the level in vesicles, and miRNA treatment has different sustainability than RNAase A treatment [14].

Cancerous cells actively release exosomes, whose composition varies according to the type of cell they came from. Exosomes derived from cancer cells have many differences in content compared to normal exosomes, especially miRNAs [15]. A study has found that cancer patients produce more exosomes than healthy individuals in their blood [16], suggesting that cancer is associated with increased exosome secretion [17]. Exosomes secreted by a tumor can bind to neighboring cells or to the extracellular matrix, or they can be passively transported through the bloodstream and other body fluids. The half-life of exosomes in the circulation is very short, and up to 90% of exosomes are removed within 5 minutes after infusion [18]. Cancer cells may insert a subset of miRNAs into exosomes through unidentified processes. Several attempts have been made in recent years to utilize exosomal miRNA as a diagnostic marker of various stages of cancer, including both early and advanced [19].

## Exosome biogenesis

Exosomes are small vesicles with a diameter of 40 to 150 nm. They are derived from the cell membrane and act as key regulators in cell–cell communication [20, 21]. First reports of exosomes in extracellular spaces were made during the late 1980s [22]. All body fluids contain exosomes produced by normal cells and pathological cells [23]. Endosomes produce exosomes [24]. Signaling pathways and cell receptors regulate exosome biogenesis [25]. A primary endocytic organelle fuses with a plasma membrane to form the early endosomes (EE) [26]. As a result of caveolin-dependent, clathrin-dependent, or independent processes involving the integration of EEs, the inclusion membrane composition, and endocytic cargo contents are shared [27]. When an EE returns the cargo molecules to the cell membrane, it becomes a "recycling endosome" (RE) or a "late endosome" (LE), also known as a multivesicular body (MVB). A late endosome can be formed from invaginated plasma membranes to form an exosome. It is after fusion with the endoplasmic reticulum and processing in the Golgi apparatus that multivesicular bodies (MVBs) are formed. After maturation, they fuse with the plasma membrane and disseminate exosomes into the extracellular environment [22, 28]. Various proteins are involved in the exosomes biogenesis, including syndecan-1, endosomal sorting complexes required for transport (ESCRT), sphingomyelinases, Sytenin-1, Rab, tumor susceptibility gene 101 TSG101, tetraspanins, apoptosis linked gene 2-interacting protein X (ALIX), phospholipids, and ceramides [29–31]. ESCRT consists of four different protein complexes including ESCRT-0, ESCRT-I, ESCRT-II and ESCRT-III. All these complexes are in close relationships, which are mediated by several specific molecules, such as vacuolar protein sorting-associated protein 4(VPS4A and VPS4B), LYST-interacting protein 5 (LIP5), Bro1 complexes (Alix, his-domain protein-tyrosine phosphatase) and BRO1 domain, and CAAX motif‐containing protein BROX [32–35]. The initial director of the ESCRT-dependent pathways in freight transportation is a checkpoint molecule ubiquitin (ub), involving all four ESCRT complexes. ESCRT0 recognizes monoubiquitinated proteins through the HRS heterodimer (STAM1/2) [36–38]. In the cytosol, HRS interacts with other proteins, including Epidermal growth factor receptor substrate 15 (Eps15) and clathrin, and classifies ubiquitinated substrates into clathrin-coated domains [36–39]. ESCRTI then binds to ESCRTII and ESCRT0, creating a solid recognition site on the endosome surface with a strong affinity for ubiquitinated cargo molecules [40]. Finally, ESCRTIII is associated with this complex, essential for membrane lysis and endosome bud secretion [41]. In the next step, the lysosomal degradation of intraluminal vesicles (ILV) initiates unless the loaded components are attacked by deubiquitinating enzymes (DUBs) [42]. Finally, the recycling of complex subunits for further activities is mediated by exposing them to ATPase VPS4 and its cofactor VTA-mediated isolation [36].

In the plasma membranes, Rab5 acts in conjunction with the effector VPS34/p150 to regulate the conversion of extracellular vesicles (EV) to LE. Endosomes recycle cargo to the cell surface for ILVs formation. ILVs could bud from the inner membrane, causing cargo separation and repositioning within the vesicles [26]. ESCRT machinery is vital for sorting proteins into intracellular compartments [36–38].

### ExomiRs in the pathogenesis of hematologic neoplasms

**Fig. 1:**
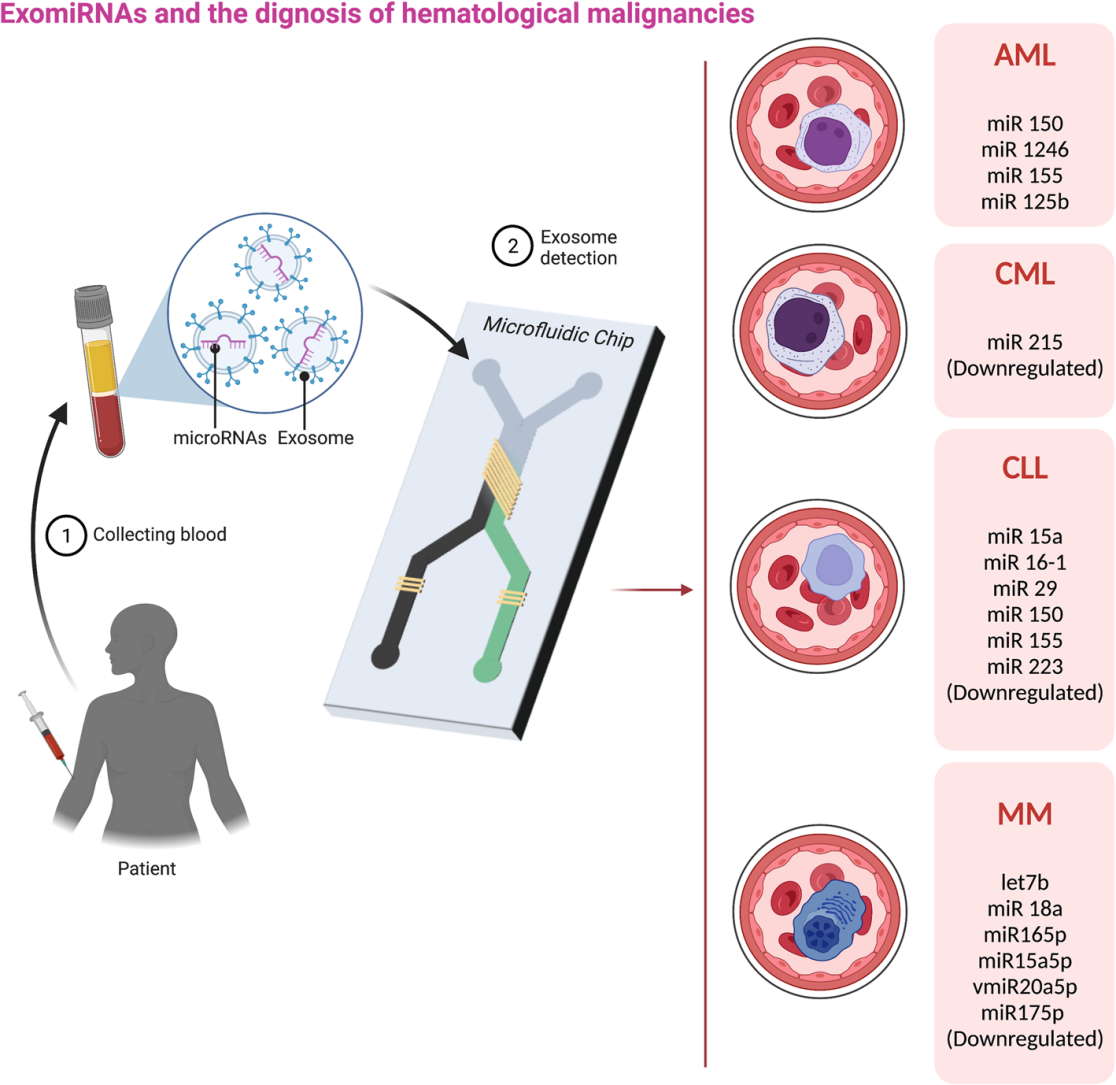
ExomiRNAs and the diagnosis of hematologic neoplasms. After the blood is obtained from the subject, it can be used for detection. The microfluidic approach is one the most accurate ways of detection (demonstrated in the figure as a microfluidic chip). These malignancies have their specific miRNA as the tumor marker. Here four disorders (AML: Acute myeloid leukemia, CML: Chronic myeloid leukemia, CLL: Chronic lymphocytic leukemia, and MM: Multiple myeloma) have been illustrated

MiRNAs are contributed to the etiology of several diseases, including cancer, and are carried as cargo by distal or adjacent recipient cells within the exosome as a way of cell-to-cell signaling that may modulate pathogenesis [43–45]. Several studies have shown that exosomes can modify gene expression in recipient cells affecting their phenotype and response. In malignancies, exosome cargos, consisting of non-coding RNAs, mRNAs, and macromolecules such as proteins, substantially affect recipient tumoral cells. With efficient exosome delivery, the miRNAs could interfere with the gene expression in target cells through binding to the mRNA sequences [46]. For a better overview, the main mechanisms of exomiRNAs in modulating pathogenesis of hematologic neoplasms is summarized in Fig. 1.

Chronic lymphocytic leukemia (CLL) patients have weakened T cell immunity. Recent studies have shown that CLL disease induces myeloid-derived suppressor cells (MDSCs), which suppress T cell activation and induce inhibitory regulatory T cells (Tregs) via exosome miR-155 metastasis [47, 48]. Transmission of miRNA to monocytes in the exosome-mediated pathway may contribute to CLL-induced immune avoidance mediated by PDL1 expression. A study on the CLL-cell line confirmed that CLL-derived exosomes contain small RNAs, and miR2023p enhances the expression of hedgehog signaling intermediates [49]. In the chronic myelocytic leukemia (CML) model, CML-derived exosome stimulated producing of interleukin (IL)-8 from bone marrow stromal cells [50, 51]. Furthermore, another study showed that exosomes secreted from CML patients' cells could directly affect the endothelium and alter angiogenic processes [52]. MM-derived exosomes can alter the microenvironment of the bone marrow to accelerate the progression of myeloma.

Conversely, stromal cells of bone marrow may jointly release specific exosomes taken up by MDSCs via the STAT1 and STAT3 pathways, increasing immunosuppression and inducing MM expansion [53]. First, researchers at the Dana Farber Cancer Institute have shown considerable profile differences in microRNA between normal and mesenchymal bone marrow stromal cells in MM [54]. MiRNA have the highest frequency of small RNAs in the MM exosomes [55]. Caspase3 was activated by stromal cell-releasing exosomes capable of cleaving the anti-apoptotic protein Bcl-xL localizes to the outer exosome membrane. By destroying Bcl-xL, these exosomes can be internalized through plasma cell myeloma and increased proliferation [56]. Researchers developed a hypoxic-tolerant MM model that mimics the hypoxic microenvironment of the inside of the body that causes rapid proliferation of MM. Their experiments showed that hypoxic-tolerant MM cells release more exosomes than parent cells under normal or acute hypoxic conditions. The major efficient protein of exosome cargo was miR135. This protein directly suppresses factor-inhibiting hypoxia-inducible factor 1 (FIH1) in endothelial cells of the bone marrow [57]. Therefore, more research is needed to test whether miR-135b can be served as a target for drugs that prevent MM angiogenesis.

### MiRNAs

MicroRNAs are a subset of non-coding RNAs (ncRNAs) that are well defined in human tissues and all types of cells [11]. The miRNAs are short, 22 nucleotides, regulatory RNA molecules that are originated from RNA precursors [58]. By binding to the 3'UTR of the target mRNA,the small RNA molecules regulate gene expression primarly at the post-translational levels. The relevant process is carried out by a ribonucleoprotein complex called the RNA-induced silencing complex (RISC) [11]. MiRNAs are able to reduce gene expression by binding to mRNA and repressing or degrading it [59]. Additionally, they transmit genetic information between cells and tissues [60]. There are thousands of genes that can be targeted by potential miRNAs. Likewise, miRNAs are suitable for regulating various pathogenical pathways [58]. The miRNAs play role in many physiological processes including apoptosis [61], hematopoiesis [62], angiogenesis [63], and metastasis [64].

A majority of miRNA biogenesis genes are located in fragile chromosomal locations or regions of the genome associated with cancer, which shows the key role of miRNAs in developing human neoplasms [65]. In addition, miRNA deregulation is involved in the cancer progression through disruption of tumor suppressors and oncogenes [65, 66]. Molecular analyzes have shown that approximately half of all human miRNAs are intragenic. Other miRNAs are synthesized by intergenic noncoding precursors [65, 67]. MiRNAs can either repress translation or degrade target mRNAs to regulate gene expression levels [68]. Both the nucleus and the cytoplasm participate in the biogenesis of miRNAs [69]. RNA polymerase II transcribes miRNA genes to primary transcripts (pri-miRNA) [70, 71]. A number of hairpin-like structures may be present in the pri-miRNAs produced. Upon processing these structures, stem-loop precursor miRNAs (approximately 70 nucleotides long) are made, which are then modified into pre-miRNAs by Drosha ribonuclease III and its binding partner DGCR8. Exportin 5 is responsible for exporting miRNAs from the nucleus to the cytoplasm [72]. Another enzyme, RNase III enzyme Dicer, and its cofactor, trans-activation response (TAR) RNA-binding protein (TRBP) further cleave pre-miRNA stem loop structure in the cytoplasm. The miRNA's guide strand enters the RNA-induced silencing complex while the other strand rapidly degrades [73]. The generated miRNA-RISC complex binds to the target 3' UTR mRNA by identifying specific binding sites. As a result, depending on how complementaristic the miRNA-mRNA linkages are, miRNA can then inhibit translation or cleave mRNA, depending on the complementarity of linkages between miRNA and target mRNA. MiRNA can then inhibit translation or cleave mRNA, depending on the complementarity of linkages between miRNA and target mRNA [74–76].

### Exosomal miRNAs in patients’ diagnosis and prognosis

An early cancer diagnosis could significantly affect the patients' survival and hint toward discovering accurate diagnostic molecules. Most of the current biomarkers in serum lack proper sensitivity and specificity for various malignancies [77, 78]. Cancer subjects and unaffected individuals express different miRNAs in their exosomes. Exosomal miRNAs can also predict tumor growth, severity, and aggressiveness and their clinical applications as cancer biomarkers are encouraging. [79].

In 2007, a study found that a subset of exosomal miRNAs in cancer patients are more accessible and with their promising diagnostic potential could replace more invasive biopsy approaches [4]. Since exosome miRNAs are highly stable, easy to access, and widely available in body fluids, they are promising as cancer biomarkers [79]. To better define whether exosome miRNAs can be used clinically as diagnostics biomarkers for predicting cancer outcomes, more research is needed [79]. Several clinical variables are associated with miRNA expression in exosomes, including tumor type and stage. Therefore, it is essential to validate the spectral use of exosome miRNAs and investigate their association with traditional cancer biomarkers.

These obstacles should be removed prior to the clinical use of exosome miRNAs: uniform techniques for isolating and detecting exosomes are not accessible, and stable established internal reference genes for precise quantification of exosome miRNAs are still under development.

Hematologic Neoplasms have diverse etiologies and prognoses, including lymphatic, myeloid, histiocytic, and mast cell neoplasms. Their prognosis is highly dependent on the pathology of the disease [80]. Current methods and techniques for diagnosing hematologic neoplasms include biopsy [81], peripheral blood test [82], bone marrow biopsy [83], immunological test [84], flowcytometry [85], and radiation. It also includes the chromosomal analysis [86] and DNA sequencing technology [87]. Because of the costly, labor-intensive, and complex features and radioactive contamination of these clinical diagnostic techniques [88], developing a new, convenient, low-cost technique with high sensitivity is being considered for diagnosing blood cancer.

Hematologic neoplasms include various types of leukemia, Hodgkin and non-Hodgkin lymphoma, and multiple myeloma. The studies around discovering the desirable tumor markers in the circulation, especially exosomal miRNAs, benefit from the circulatory nature of hematologic neoplasms [89, 90]. Honick et al., using a rodent model of acute myeloid leukemia (AML), identified that miR150, miR1246, and miR155 are rich in murine serum exosomes. The researchers proposed these three exosome miRNAs as the primary serological biological markers for early diagnosis of AML [91]. In another study, circulatory exosomes harboring miR125b were evaluated as a prognostic factor in subjects with moderate risk of AML. It was concluded that elevated levels of exomiR125b was linked to higher recurrence rates and complications [92]. In addition, in chronic myeloid leukemia (CML), downregulated exosome miR-215 levels are significantly lower in CML patients with minimal residual disease, so exosome miR-215 levels are untreated manuscripts. It has been shown that it can be accepted as information to reach. Diagnosed as discontinuation of treatment with imatinib [93]. MiR-15a and miR-16–1 are eliminated or downregulated in the most CLL cells [94–96], and re-expression of these miRNAs induces apoptosis by downregulation of BCL2. For CLL, analysis of plasma-derived exosomes determined CLL exosome miRNA profiles such as downregulation of miR-29 (miR29a/b/c), miR-150, miR-155, and miR-223. In addition, B-cell receptor signaling increases exosome production and miR-150 and miR-155 expression and is contraindicated in treatment with ibrutinib [97]. The most common malignant blood disease in Western countries is non-Hodgkin’s lymphoma; the second is multiple myeloma, accounting for 13% of blood cancers. A study that surveyed circulating exosome miRNAs in serum of 156 MM patients identified that 22 miRNAs had significantly Lower expression in MM subjects than in unaffected controls. Among them, let7b and miR-18a were significantly correlated with survival. Patients with low exosome levels of let7b and miR-18a have poor outcomes [55]. In the age of personalized medicine, drug resistance prediction tools are becoming more critical. Zhang et al., focus on predictable values ​​of exosome miRNAs that cause drug resistance in MM patients. They considered data from 204 patients and found that the exosomes miR-165p, miR-15a5p and, vmiR-20a5p, miR-175p were downregulated in bortezomib-tolerant patients [98].

Secretion of exosome miRNA from different cell types present in the tumor microenvironment may regulate immune responses in the tumor microenvironment. Exosomes with miR-9 secreted by tumor cells have been shown to repress major histocompatibility complex (MHC) class I expression and prevent the immune system from identifying tumor cells [99]. Macrophages are important host immunomodulators, and tumor-associated macrophages (TAMs) exert immunosuppressive effects by secreting cytokines that suppress antitumor responses. Fabbri M. et al., Publication showed that exosome-derived miRNAs bind to this human Toll-like receptor 8 (TLR8). It has the same functionality as the mouse TLR7 [100].

The binding of exosome-derived to these receptors triggers downstream activation of NF-κB signaling and the production of inflammatory mediators in macrophages [46]. In this regard, exosome-derived miR-21 that bind to TLRs on macrophages have been linked to inducing the expression of inflammatory cytokines and promoting tumor migration [100].

**Fig. 2:**
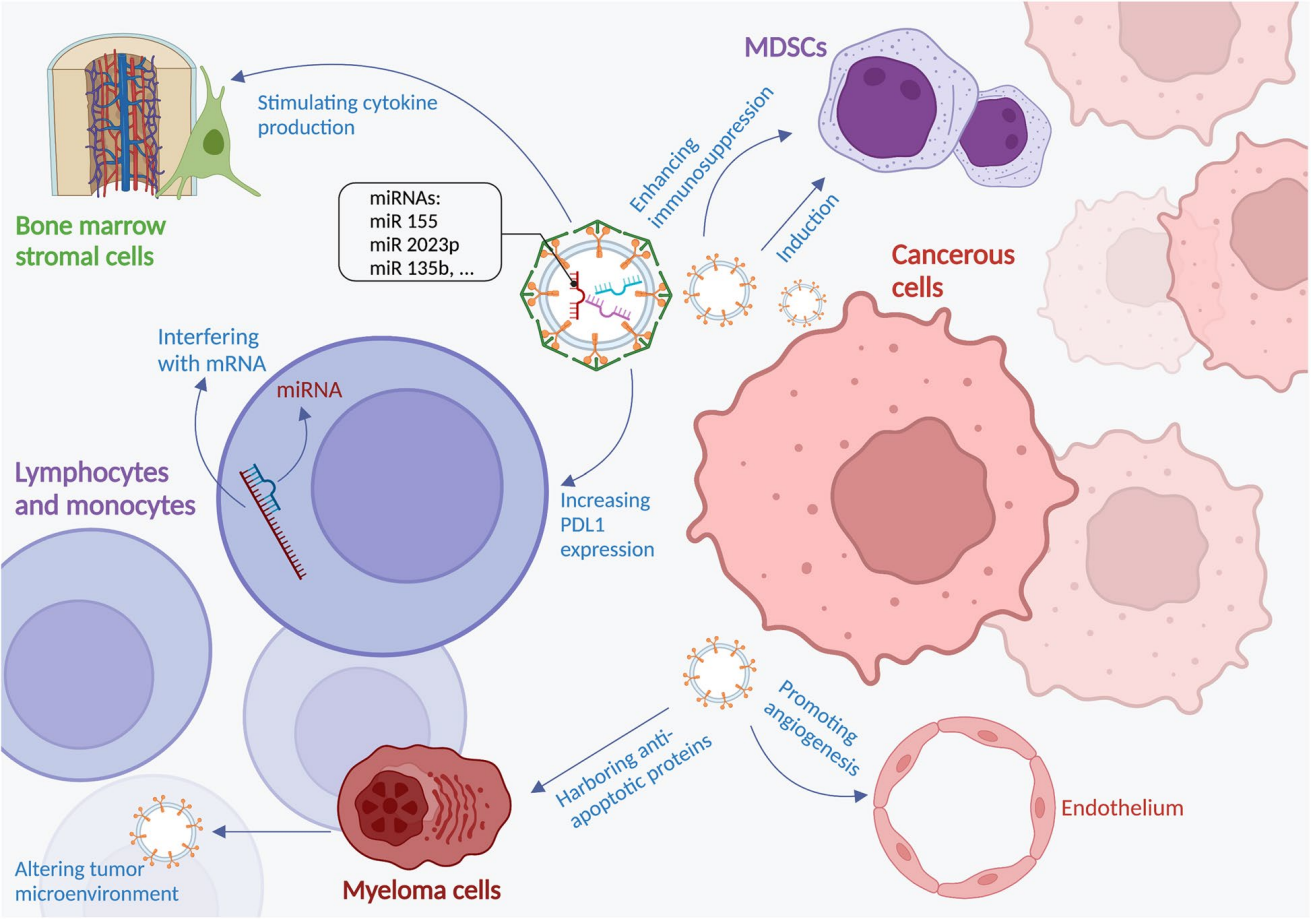
The role of exomiRNAs in the pathogenesis of hematologic neoplasms**.** Cancer cells could release exosomes filled with miRNAs with mRNA-interfering potential in target cells. The miRNAs could also harbor antiapoptotic proteins and promote angiogenesis along with improving the immunosuppressive function of myeloid-derived suppressor cells (MDSCs) and manipulating the tumor microenvironment

The potential of exomiRNAs in developing biomarkers and diagnostics of hematologic neoplasms are illustrated in Fig. 2.

### Therapeutic roles of exomiRs

In addition to exomiRNAs potential for early detection and possible diagnostics implications, they are candidates for developing modern personalized treatment tools and follow-up modalities. Currently, the clinical significance of circulating miRNAs in predicting treatment responses and outcomes in patients with a wide range of human malignancies has been documented [101]. It has been identified that circulating exo-miRNA is a possible diagnostic and a prognostic biomarker in the cancer treatment monitoring [102]. One strategy for treating exosomes is to inhibit oncomiR expression by delivering antagonistic tumor-suppressing miRNAs to treat cancer. Exosomes loaded with therapeutic anti-miRNA oligonucleotides complementary to the mature carcinogenic miRNAs sequences can be delivered by local injection or systemic delivery into the tumor. Another therapeutic approach to inhibit tumor genesis is to remove exosomes from the circulatory system or avoid exosome fusion or absorption to target cells. Exosomes are isolated from the patient's body fluids, corrected, and then transferred to the same patient for targeted cancer treatment. Exosomes utilize for liquid biopsy since it’s not invasive. Exosomes are ideal candidates for predicting treatment response in melanoma patients by increasing PD-1 and CD28 expression in exosomes. It is possible to isolate exosomes from patients and engineer them by lectins or antibodies against CD63, CD81 which are exosome markers or manipulate them and after modification, transfer them back to the patients. Exosomes play roles in drug delivery: exosomes isolated from different cell types are rich in miRNA, RNA and protein. These molecules can further modified and reinserted into the exosomes for different therapeutic applications [103–105]. In addition, exosomes that deliver some miRNAs may be ideal candidates for inhibiting tumor growth using specific gene knockdowns. Exosomes are future biomarkers in medicine and highly effective "nanovectors" as delivery vehicles for targeted anti-cancer agents that are less immunogenic and less toxic than other drug delivery tools in the anti-cancer therapy [106, 107]. Because exosomes are small, non-toxic, non-immunogenic, and membrane junctions, they are endemic to humans. Drug cargos in exosome-based vehicles can overcome the blood–brain barrier, allowing the delivery of essential therapeutics to the brain. Isolation of milk exosomes was done, and they were loaded with Withaferin, an anticancer drug, and used in mouse models of breast and lung cancer [108]. Engineering design allows miRNAs, siRNAs, genes, small molecule reactive biomolecules, peptides, and antioxidants to load into the exosome [109].

**Fig. 3:**
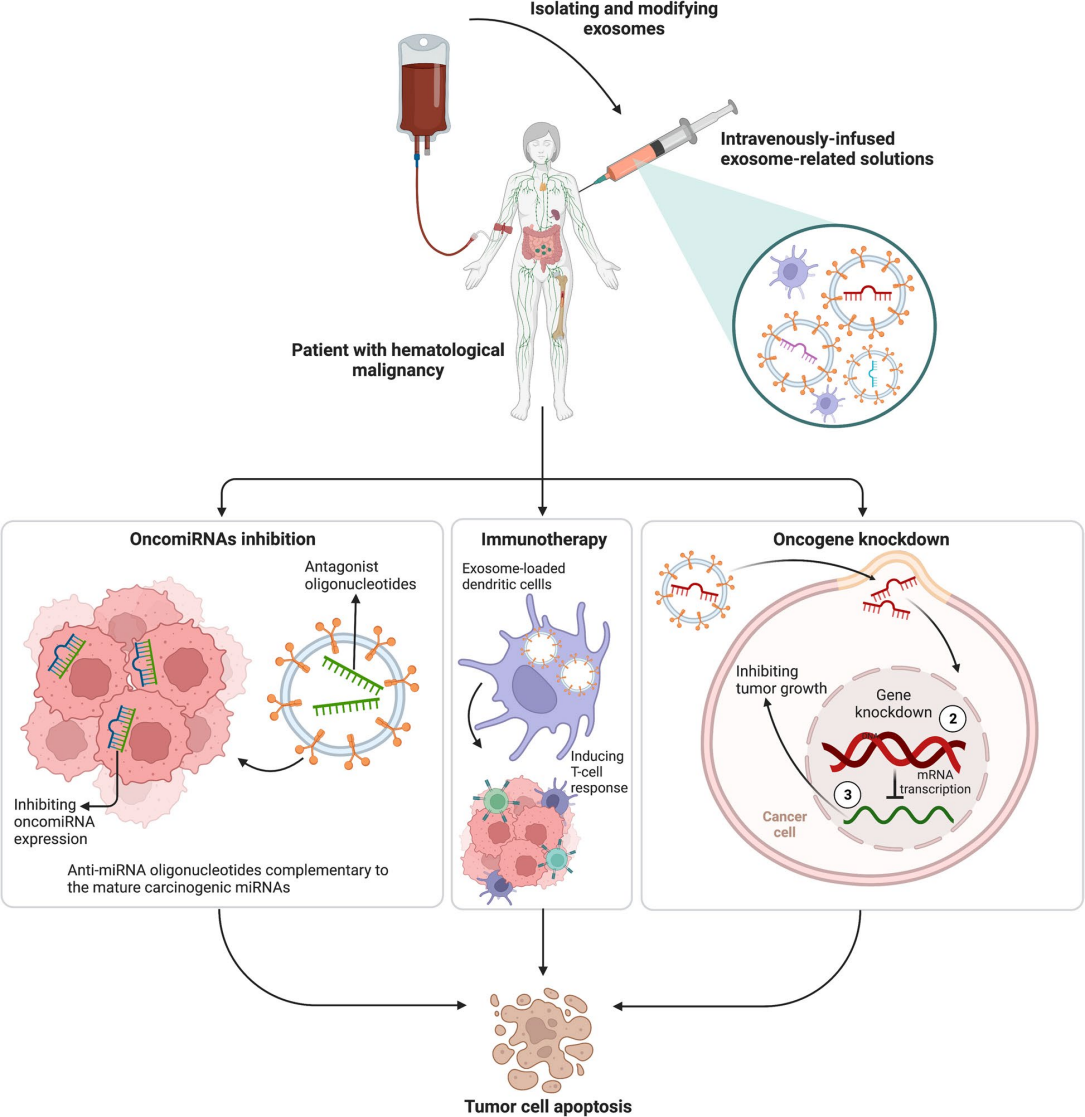
Different approaches for treating hematologic neoplasms through exosomes. Blood is drawn from the patient and exosomes will be isolated and then modified. Modified exosomes with specific miRNA and oligonucleotides cargos either obtained from patient or synthesized could be locally injected or systemically delivered. These cargos could 1) Inhibit oncomiRNAs in cancer cells by complementary oligonucleotide attachment, 2) Induce robust T cell response against tumors via dendritic cells (DCs) filled with modified exosomes, and 3) Suppress tumor growth by knocking out the target oncogene

Presently, several exosome-based clinical trials are being conducted to treat cancers worldwide. However, the clinical use of exosomes to solve many controversial problems requires further research and appropriate validation [110, 111]. The theory was the use of exosomes in immunotherapy. First, it was proposed in 1996 and has recently been considered for the exosome-based immunotherapy [112, 113]. Exosomes and DCs pulsed with Tumor-derived exosome (TDE) isolated from cancerous cells have been investigated for their antitumor properties [114]. We have shown that interactions between Montecarbo and other DCs occur via exosome miRNAs. Exosome content can also change during the parental DC maturation [115]. No NK cells or T cells were activated by DC immunostimulatory activity. DC decreases the immunosuppressive function of Tregs and MDSCs; however, using DC for treatment may have a detrimental effect on these cells [116]. The different therapeutic approaches of miRNA using exosomes are illustrated in Fig. 3.

Allogeneic stem cell transplantation (SCT) kills leukemic cells and improves AML patients. Consequently, adoptive immunotherapy with alloreactive NK cells will be a strategy in AML treatment [117]. Activated NK cells produce vast amounts of exosomes, which are absorbed by K562 cells and exert exosome anti-leukemic activity against AML cells in a dose-dependent manner [118]. Phosphatase and Tensin Homolog (PTEN) is a tumor suppressor as an antagonist of the PI3K-Akt signaling pathway, and miR-21 has been reported to increase PTEN infiltration and proliferation [119]. Studies show that curcumin treatment increases PTEN in a dose-dependent manner, reducing AKT phosphorylation and vascular endothelial growth factor **(**VEGF) expression. In addition, reduced levels of miR-21 are observed in CML cells, amplified after treatment with curcumin in exosomes. Experiments with mouse models also showed that mice treated with curcumin had smaller tumors than untreated mice [120, 121].

The immunosuppressive effect of TDE is due to miRNAs that can be used to follow up patients. For example, mutual detection of miR-150, 155, and 1246 in AML-derived electric vehicles have been suggested in this context [91]. New treatment approaches have replaced current cancer treatments due to their low success rate and painful side effects such as chimeric antigen receptor (CAR) expressing exosomes. The new therapeutic approaches have few side effects [122].

The biological properties of electric vehicles make them ideal for drug delivery systems with few side effects, mainly because they have few side effects [123]. Proteomics analysis of electric vehicles has suggested that it may help suppress undesirable side effects such as the spread of cancer genes, prions, inflammatory cytokines, or virus particles. The minimal side effects associated with DC-derived exosomes' antitumor effects are a significant feature of these exosomes [124, 125].

As another type of vesicle, cell surface vesicles have been shown to play an important role in biology. Known EVs of these cell membrane vesicles can also reduce the side effects of the drugs such as doxorubicin compared to the control group [126]. According to these studies, using exosomes has important consequences for reducing side effects in treating malignant tumors. Considering today's importance of this topic, it is precious to understand its various aspects [126].

## Conclusion

Exosomes are cutting-edge tools featuring distinct capabilities as the extra-cellular vesicles ranging from effective cargo delivery to implications in developing novel biomarkers. Among their cargos, miRNAs have recently gained substantial attention for their role in various cellular signaling pathways contributing to disease pathogenesis. The miRNAs are promising biomarkers for cancer diagnostics and prognostics and play a key role in cancer progression. In hematologic neoplasms, different miRNAs have been identified in the development, early detection, and possible treatment of these disorders. In this review, we discussed the recent evidence around the role of exosomal miRNAs in these categories and highlighted their potential. Exosomal miRNAs can also provide a wide range of information about tumor stage, tumor progression, and tumor classification or subtype. However, several barriers should be addressed before they could be implicated in clinical settings. The top issues are the lack of standard techniques for isolating and detecting exosomes and appropriate methods for selecting reference genes. Future research could shed light on a more applicatory approach to exosomal miRNAs in gene targeting and immunotherapy of hematologic neoplasms.   

## Data Availability

Not applicable.

## Abbreviations

CAR-T cell: Chimeric antigen receptor T cell
EV: Extracellular vesicles
MDSC: Myeloid-derived suppressor cell
MM: Multiple myeloma
ncRNAs: Non-coding RNAs
SCT: Stem cell transplantation
TDE: Tumor-derived exosome
VEGF: Vascular endothelial growth factor
PTEN: Phosphatase and TENsin Homolog
